# PCSK9 in Vascular Aging and Age-Related Diseases

**DOI:** 10.14336/AD.2024.1713

**Published:** 2025-02-27

**Authors:** Dong Tan, Xin Yang, Jing Yang, Gang Fan, Guozuo Xiong

**Affiliations:** ^1^Department of Vascular Surgery, the Second Affiliated Hospital of University of South China, Hengyang, Hunan, China.; ^2^Pan-Vascular Research Group, Shenzhen University Affiliated Sixth Hospital, Shenzhen, Guangdong, China.; ^3^Department of Urology, Shenzhen University Affiliated Sixth Hospital, Shenzhen, Guangdong Province, China.; ^4^Department of Metabolism and Endocrinology, the First Affiliated Hospital, Hengyang Medical School, University of South China, Hengyang, Hunan, China.; ^5^Hunan Province Thrombotic Disease Prevention and Treatment Clinical Medical Research Center, The Third Affiliated Hospital of University of South China, Hengyang, Hunan, China.; ^6^Hunan Province Thrombotic Disease Prevention and Treatment Clinical Medical Research Center, The Second Affiliated Hospital of University of South China, Hengyang, Hunan, China

**Keywords:** PCSK9, vascular aging, age-related diseases

## Abstract

The aging process significantly contributes to human disease, and as worldwide life expectancy increases, addressing the challenges of aging and age-related cardiovascular diseases is becoming increasingly urgent. Vascular aging is a key link between aging and the development of age-related diseases. Recent studies indicate that proprotein convertase subtilisin/kexin type 9 (PCSK9), a type of protein involved in the metabolism of lipids, is crucial in modulating vascular aging by affecting the physiological functioning of vascular cells. PCSK9 is linked to lipid metabolism and chronic inflammation and is involved in regulating senescence-related activities, including migration, proliferation, apoptosis, and differentiation. These factors contribute to the aging of vascular cells and age-related vascular diseases, including atherosclerosis, hypertension, coronary artery disease, and cerebrovascular diseases. Given its involvement in these processes, this article provides a comprehensive summary of PCSK9's regulatory functions in vascular aging, highlighting potential therapeutic targets for combating age-related cardiovascular diseases.

## 1. Introduction

As a growing concern globally due to advancements in medical science and longer life spans, aging is an inevitable biological phenomenon and an independent risk indicator for numerous chronic diseases, such as cardiovascular disease [1]. Cardiovascular diseases are circulatory disorders resulting from cardiac abnormalities and vascular dysfunction, which is the principal cause of mortality worldwide, constituting almost one-third of all deaths [2-6]. Vascular aging involves alterations in the structure and function of the arterial wall as one age. Morphologically, it involves increased collagen fibril accumulation, disorganized elastic fibers, and thickening of the inner layer. This leads to increased stiffness in the media and intima layers, decreased elasticity, reduced availability of nitric oxide, leading to endothelial dysfunction, and an impaired ability to regulate blood flow in reaction to heightened metabolic demands. Vascular aging can result in persistent arterial inflammation, modified angiogenesis, and structural changes due to alterations in vascular cell function, thereby elevating the likelihood of vascular age-related disorders [7-9]. Understanding the causes of arterial aging is crucial, as it contributes to the onset and progression of age-related cardiovascular and cerebrovascular diseases. This will enable the development of anti-aging intervention strategies that prevent and manage chronic cardiovascular diseases in the elderly, thereby addressing an issue that is becoming increasingly critical [10].

PCSK9, initially once referred to as neural apoptosis-regulated convertase 1, was first discovered in cerebellar neurons undergoing apoptosis [11]. Many studies have now made it clear that PCSK9 binds to the liver low-density lipoprotein receptors (LDLR), which causes them to be taken inside together and makes it easier for cells to destroy the receptor. This shows that PCSK9 has a role in hyperlipidemia and is linked to atherosclerosis, a chronic degenerative vascular disease [12]. Aging is correlated with disruptions in cholesterol metabolism, with emerging evidence suggesting that PCSK9 is involved in various metabolic pathways associated with vascular aging [13, 14]. However, the influence of PCSK9 on the aging process remains largely uncharted and necessitates additional research. Comprehending the involvement of PCSK9 in aging could provide crucial perspectives for potential therapeutic strategies targeting age-related cardiovascular conditions. This paper aims to provide a concise overview of the biological characteristics of PCSK9, its mechanism for functioning in the vascular system, especially in vascular aging. The pertinent research on PCSK9 in diseases associated with vascular aging was also summarized. And the potential for therapy of PCSK9 inhibitors in treating vascular cell senescence and senescence-related vascular disease.

## 2. Biological characteristics of PCSK9

In 2003, Nabil Seidah and Jae Byun identified a novel human proprotein convertase, with its gene situated on the short arm of chromosome 1. And the first identification of functional mutations in the PCSK9 gene leading to dominant familial hypercholesterolemia (ADH) provided valuable insights into the physiological function of PCSK9 [15].

PCSK9 constitutes the last member of a serine protease family, which bears a closer resemblance to bacterial subtilisin [16]. Enzymes belonging to this particular family possess distinct physiological functions and are involved in the activation or deactivation of diverse proteins through processing or regulation [17]. The initial eight convertases are responsible for the cleavage of secretory protein precursors, resulting in the production of fully developed, functional, and biologically active peptides and hormones that provide a vital function in the control of development and metabolism [18]. However, PCSK9 undergoes self-cleavage and subsequently stops functioning as a protease. Instead, it operates in a nonenzymatic manner to facilitate the degradation of the LDLR to control the liver's apolipoprotein B (apoB) lipoprotein absorption and cholesterol metabolism [19]. PCSK9 acts as a serine protease that functions via the LDLR to control the liver's apoB lipoprotein absorption and cholesterol metabolism [20]. The LDLR pathway is primarily responsible for the removal of plasma low-density lipoprotein cholesterol (LDL-C) from the plasma. Following their binding, low-density lipoprotein (LDL) and LDLR are absorbed into clathrin-coated pits and subsequently subjected to lysosomal degradation. LDLR is then returned to the cell membrane by recirculation [21]. PCSK9 binding to the cell surface LDLR, specifically through the LDLR epidermal growth factor (EGF)-like domain, triggers LDLR internalization. PCSK9 inhibits the conformational shift of LDLR, which causes LDLR to be redirected to a lysosome for degradation and reduces LDL-C clearance from the plasma [22].

Interestingly, recent studies have revealed that PCSK9 is secreted by various cell types, including vascular endothelial cells, smooth muscle cells, and macrophages, and its biological roles extend beyond the regulation of circulating LDL cholesterol levels [23-25]. Its secretion is intricately linked to inflammatory processes, as PCSK9 has been demonstrated to stimulate pro-inflammatory cytokine production in macrophages and liver cells, thereby establishing a connection between PCSK9 and the inflammatory milieu associated with atherosclerosis [26]. At a molecular level, PCSK9 interacts with toll-like receptor 4 and regulates nuclear factor kappa-light-chain-enhancer of activated B cells (NF-κB) activation, influencing apoptosis and autophagy pathways [27]. This multifaceted role suggests that PCSK9 is not merely a cholesterol regulator but also a key player in the inflammation-driven mechanisms of cardiovascular disease. The association between PCSK9 and inflammation provides a compelling rationale for the therapeutic use of PCSK9 inhibitors in preventing atherosclerosis and its severe consequences, such as myocardial ischemia.

In addition, in vascular smooth muscle cells and human atherosclerotic plaques, the high expression of PCSK9 is regulated by nicotinamide adenine dinucleotide phosphate (NADPH) oxidase-derived reactive oxygen species (ROS), which promote the formation of oxidized low-density lipoprotein (ox-LDL) [28]. Ox-LDL, in turn, can induce the overexpression of PCSK9, further advancing the progression of atherosclerosis, and thereby establishing a positive feedback loop that promotes atherosclerotic development [29]. Research indicates that low shear stress increases PCSK9 expression via facilitating the production of ROS in vascular ECs and VSMCs. Reactive oxygen species seem to influence the regulation of PCSK9 expression [24]. Moreover, the overexpression of the PCSK9 gene, generated by plasmid transfection, increases ROS generation in macrophages derived from knockout mice [30]. PCSK9 inhibitors (PCSK9i) possess inherent antioxidant properties in endothelial cells, which are at least partially mediated by SIRT3 [31]This suggests that PCSK9 inhibition could be a new approach to reduce oxidative stress-related cardiovascular risks.

The PCSK9 promoter is subject to complex regulatory mechanisms involving various transcription factors, notably the sterol response located in binding proteins, which bind to the sterol regulatory element within the promoter region [32]. Low cholesterol levels stimulate sterol regulatory element-binding protein-1 (SREBP-1) and SREBP-2 expression, facilitating an increase in circulating PCSK9 levels [32, 33]. Other transcription factors, such as hepatocyte nuclear factor 1 alpha (HNF1α) and forkhead box O3 (FoxO3), further modulate PCSK9 expression, highlighting the intricate interplay of metabolic signals in PCSK9 regulation [34]. Notwithstanding considerable progress in our comprehension of PCSK9 biology, the regulation of its expression and secretion remains dynamic and complex. Ongoing research is poised to elucidate the broader implications of PCSK9 interactions with other receptors and pathways in cardiovascular biology, paving the way for novel therapeutic strategies targeting this multifaceted protein [35].

## 3. Role of PCSK9 in vascular aging

**Figure 1.**
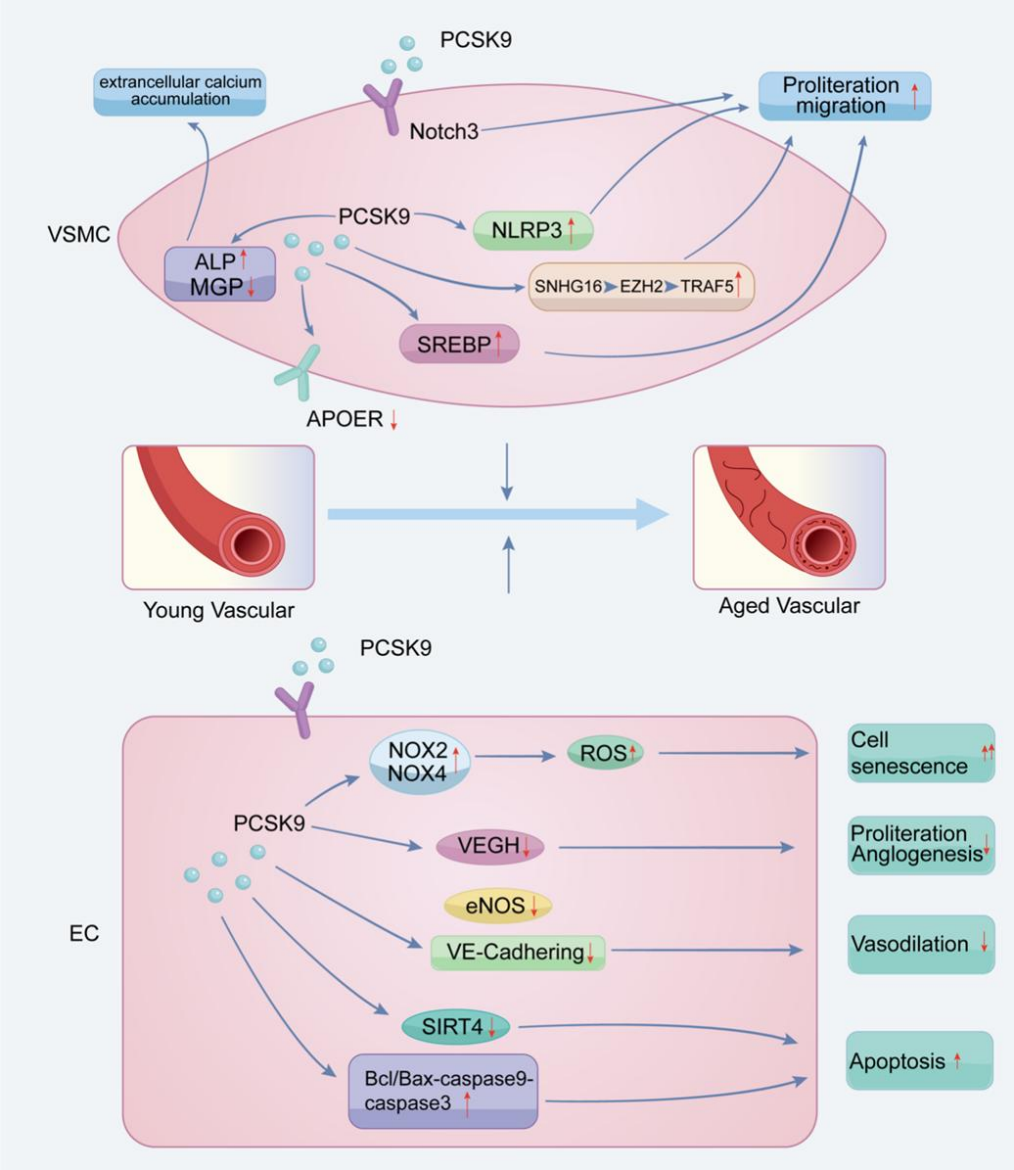
**The Role of PCSK9 in Vascular Aging**. In VSMCs, PCSK9 promotes proliferation and migration by activating the Notch3 receptor, NLRP3, and SREBP, as well as through the SNHG16/EZH2/TRAF5 axis. PCSK9 reduces MGP expression and increases ALP levels, leading to extracellular matrix calcification. Additionally, it induces cellular senescence by downregulating APOER. In ECs, PCSK9 decreases VEGF levels, thereby impairing migration and angiogenesis. It reduces eNOS and VE-cadherin expression, affecting endothelium-mediated vasorelaxation. PCSK9 activates the Bcl/Bax-caspase9-caspase3 pathway to enhance apoptosis. Furthermore, it increases ROS production via NOX2 and NOX4 and downregulates SIRT4, contributing to cellular senescence. Abbreviations: VSMCs: vascular smooth muscle cells; NLRP3: NOD-like receptor family pyrin domain containing 3; SREBP: sterol regulatory element-binding protein; SNHG16: small nucleolar RNA host gene 16; EZH2: enhancer of zeste homolog 2; TRAF5: TNF receptor-associated factor 5; MGP: matrix GLA protein; ALP: alkaline phosphatase; APOER: apolipoprotein E receptor; ECs: endothelial cells; VEGF: vascular endothelial growth factor; eNOS: endothelial nitric oxide synthase; VE-cadherin: vascular endothelial cadherin; ROS: reactive oxygen species; NOX2: nicotinamide adenine dinucleotide phosphate Oxidase 2; NOX4: nicotinamide adenine dinucleotide phosphate Oxidase 4; SIRT4: Sirtuin 4.

The role of PCSK9 in vascular aging is increasingly recognized as a critical area of research, given its multifaceted influence on vascular biology [33, 36, 37]. PCSK9 is conventionally recognized for its role in lipid metabolism and atherosclerosis; however, emerging evidence suggests that it also plays a pivotal role in the processes underlying vascular cell senescence, inflammation, and calcification, which are integral to vascular aging [13, 14, 38]. Cellular senescence, characterized by irreversible growth arrest, is influenced by various stressors, including oxidative damage and inflammation, both of which are modulated by PCSK9 [39, 40]. Research has shown that PCSK9 can induce apoptosis in vascular endothelial cells (ECs) and smooth muscle cells (VSMCs) through multiple signaling pathways, thus contributing to vascular cell senescence [41, 42]. Furthermore, PCSK9's interactions with proinflammatory cytokines and reactive oxygen species underscore its role in promoting vascular inflammation, a key factor in the progression of vascular aging [43]. Additionally, the upregulation of PCSK9 in pathological conditions leads to mechanisms that favor vascular calcification, further complicating its role in vascular health [38]. This discussion will delve into the specific mechanisms by which PCSK9 influences vascular cell senescence, cellular proliferation, migration, and apoptosis, as well as its implications for vascular inflammation and calcification within the framework of vascular aging (Fig. 1).

### 3.1 PCSK9 and cellular senescence

Cellular senescence, particularly in vascular ECs and VSMCs, has emerged as a critical mechanism underlying age-related cardiovascular diseases [44]. The senescence of ECs is intricately associated with oxidative stress, primarily mediated by ROS such as hydrogen peroxide and peroxynitrite [45]. Nicotinamide adenine dinucleotide phosphate (NADPH) oxidase is a significant contributor to ROS production, and the resultant oxidative damage fosters EC senescence, characterized by increased pro-inflammatory cytokine release, including interleukin-1 beta (IL-1β), interleukin-6 (IL-6), and tumor necrosis factor-alpha (TNF-α), through the activation of the nuclear factor kappa-light-chain-enhancer of activated B cells (NF-κB) signaling pathway [46, 47]. Importantly, defective efferocytosis—the clearance of apoptotic cells—exacerbates inflammation and contributes to the senescent phenotype in ECs, a process further influenced by increased ROS production and mitochondrial dysfunction [48, 49].

An increasing amount of evidence associates PCSK9 with the regulation of cellular senescence. Recent studies indicate that PCSK9 promotes ROS generation, induces mitochondrial deoxyribonucleic acid (DNA) damage, and activates the NOD-, LRR-, and pyrin domain-containing protein 3 (NLRP) inflammasome, thereby impairing efferocytosis and potentially accelerating EC senescence [14, 33]. Furthermore, PCSK9's role extends to VSMCs, where it is highly expressed [50]. VSMC senescence, driven by factors such as oxidative stress and inflammation, leads to plaque instability and impaired vascular repair mechanisms [51]. The plasticity of VSMCs allows them to shift between contractile and synthetic phenotypes under pathological conditions, a transition elevatedly influenced by PCSK9 expression [13]. Notably, the upregulation of PCSK9 in atherosclerotic plaques and ischemic tissues suggests a direct contribution to vascular aging and cardiovascular pathology.

Emerging research highlights a complex interaction between PCSK9 levels and cardiovascular aging. Notably, a comprehensive study by the Pacher laboratory demonstrated a marked increase in plasma PCSK9 levels associated with aging, underscoring its potential role in cardiovascular dysfunction [52]. Coupled with findings from hemodynamic analyses in translational models, these data suggest that elevated PCSK9 not only correlates with but may also drive age-related cardiovascular changes, including mitochondrial dysfunction, oxidative stress, and fibrotic remodeling [50, 53]. Inhibition of PCSK9 has surfaced as a possible treatment strategy. Chronic treatment with PCSK9 inhibitors in aging models has been demonstrated to reinstate and restore LDL receptor levels, reduce circulating oxidized LDL, and mitigate pathological cardiac changes associated with aging [36, 54]. These findings suggest that targeting PCSK9 could ameliorate both lipid-related and broader age-related cardiovascular risks.

Despite the compelling evidence linking PCSK9 to cardiovascular aging, the specific mechanisms by which it influences VSMC and EC senescence remain to be fully elucidated. Moreover, the potential for direct effects of PCSK9 on cardiac and vascular tissues warrants further investigation. As the field progresses, understanding the intricate relationships between PCSK9, cellular senescence, and cardiovascular aging will be pivotal in developing effective therapeutic interventions for age-related cardiovascular diseases (Fig. 1).

### 3.2 PCSK9 and cellular proliferation, migration, and apoptosis

VSMC proliferation and migration are fundamental processes in vascular development, tissue regeneration post-vascular injury, diffuse intimal thickening in age-related vascular diseases, and maladaptive vascular remodeling associated with vascular aging [7]. Previous research indicates that PCSK9 has an impact on the proliferation rate and migratory capacity of primary VSMCs by using Pcsk9^-/-^ and Pcsk9^+/+^ mice [55, 56]. The overexpression of PCSK9 has been shown to enhance the migration, proliferation, and phenotypic transition of homocysteine-induced VSMCs [57]. PCSK9-deficient smooth muscle cells exhibited impaired proliferation and migratory abilities, which was linked to the prevention of NOD-like receptor family pyrin domain containing 3 (NLRP3) inflammasome activation [56, 58]. Lupo and colleagues engineered rat smooth muscle cells to overexpress human PCSK9, resulting in accelerated cell growth and heightened activity of sterol regulatory element-binding protein (SREBP), a protein involved in cholesterol synthesis [56]. Additionally, the PCSK9-Notch3 signaling pathway was identified as crucial for the proliferation and migration of PASMCs [59]. A PCSK9 inhibitor was found to mitigate atherosclerosis by modulating the small nucleolar RNA host gene 16 (SNHG16)/enhancer of zeste homolog 2 (EZH2)/TNF receptor-associated factor 5 (TRAF5) axis, thereby restraining the migration, proliferation and formation of foam cells by VSMCs [60]. These findings suggest that PCSK9 may contribute to vascular remodeling by promoting the proliferation, and migration of VSMCs and may also contribute to the regulation of vascular aging.

PCSK9, a known contributor to atherosclerosis, has been identified as contributing to cellular migration. Within a plaque, macrophages releasing inflammatory cytokines have been shown to promote the multiplication and movement of VSMCs in the media layer, thereby furthering the progression of atherosclerosis [61]. In a recent study, exposure of macrophages to 2.5µg/ml PCSK9 for 24 hours resulted in elevated levels of IL-6, IL-1β, TNF-α, C-X-C motif chemokine ligand 2 (CXCL2), and monocyte chemoattractant protein-1 (MCP1) mRNA. This upregulation of inflammatory mediators led to an accelerated inflammatory response [62].

Endothelial activity and proliferation capacity provide a pivotal function in the repair of vascular damage [63]. The reduction in angiogenesis and reparative capacities signifies vascular aging. [10]. Interestingly, PCSK9, recognized for its function in lipid metabolism, may also be crucial in angiogenesis. In a study, evolocumab, an inhibitor of PCSK9, significantly enhanced the vitality of human umbilical vein endothelial cells (HUVECs), leading to improved cell migration, tubule length, size, and number of junctions. Moreover, administration of evolocumab resulted in increased release of vascular endothelial growth factor (VEGF) into the supernatants of HUVECs [64]. Furthermore, a PCSK9-inhibiting antibody has been shown to enhance the ability of human high-density lipoprotein (HDL) to reduce ROS production in endothelial cells and promote cell migration [65].

Inhibition of PCSK9 has been linked to increased expression of endothelial nitric oxide synthase (eNOS) and vascular endothelial cadherin (VE-cadherin), which contribute to the maintenance of endothelium-dependent vasodilation and improved survival rates in septic mice [66]. Moreover, treatment with a PCSK9 inhibitor increased Sirtuin 4 (SIRT4) protein levels, mitigating inflammatory processes such as pyroptosis and autophagy, ultimately preventing vascular damage [67]. A study involving patients with ST-elevation myocardial infarction revealed a strong association between PCSK9 levels and endothelial dysfunction, assessed through the rate of apoptosis in HUVECs [68].

Inclisiran, another type of PCSK9 inhibitor, has been shown to suppress pyroptosis and reduce levels of cleaved-caspase-1 and NLRP3, as well as prevent cell death induced by ox-LDL in HUVECs [65]. Additionally, PCSK9 deficiency has been linked to reduced levels of increased anti-apoptotic proteins and pro-apoptotic proteins in HUVECs, inhibiting endothelial cell death via the MAPK signaling pathway [69].

Research suggests that PCSK9 siRNA may suppress ox-LDL-induced apoptosis in HUVECs by impacting the Bcl/Bax-caspase9-caspase3 pathway, thereby reducing the Bcl-2/Bax ratio and inhibiting caspase9 and caspase3 activation [70]. Furthermore, a positive feedback loop between scavenger receptors like lectin-like oxidized low-density lipoprotein receptor-1 (LOX-1) and PCSK9 has been identified, leading to mitochondrial reactive oxygen species (mtROS) production, worsening cellular damage, and ultimately cell death [71, 72] (Fig. 1).

**Figure 2.**
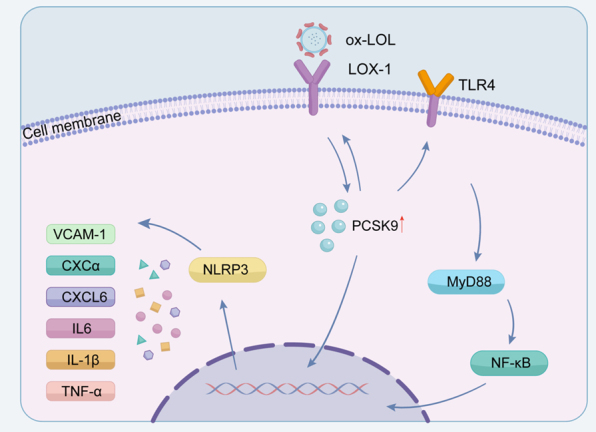
**The Role of PCSK9 in Inflammation and its Potential Impact in Aging.PCSK9 activates the TLR4/NF-κB pathway and also upregulates expression levels through its interaction with LOX-1,which ultimately leads to increased NLRP3 expression**. This process promotes the production of various inflammatory factors, including VCAM-1, CXCL6, IL-6, IL-1β, and TNF-α. Abbreviations: ox-LDL :oxidized low-density lipoprotein; TLR4: toll-like receptor 4; NF-κB: nuclear factor kappa-light-chain-enhancer of activated B cells; LOX-1: lectin-like oxidized low-density lipoprotein receptor-1;VCAM-1: vascular cell adhesion molecule-1; CXCL6: C-X-C motif chemokine ligand 6; IL-6: interleukin-6; IL-1β: interleukin-1β; and TNF-α: tumor necrosis factor-alpha.

### 3.3 PCSK9 and vascular inflammation

Chronic, sterile, low-grade inflammation is widely acknowledged as the primary factor contributing to aging, supported by convincing experimental and clinical evidence [73]. Age-related changes in blood vessels can cause long-lasting inflammation in the arteries, which can subsequently facilitate the advancement of vascular aging and related disorders. [74].

PCSK9 regulates vascular inflammation, and its expression is associated with the release of pro-inflammatory cytokines, recruitment of inflammatory cells, and instability of plaques (Fig. 2) [75]. Lectin-like oxidized low-density lipoprotein receptor-1 (LOX-1) is an established mediator of inflammation and atherosclerosis [76]. Research has demonstrated a beneficial interaction between PCSK9 and LOX-1 in VSMCs, where LOX-1 activation triggers the production of PCSK9. PCSK9 also stimulates LOX-1 production, facilitates the uptake of ox-LDL, and hence leads to a pro-inflammatory condition [71]. The toll-like receptor 4 (TLR4)/NF-κB signaling pathway is a crucial pathway affected by PCSK9, resulting in an upregulation of pro-inflammatory cytokines [77]. Recent research conducted by Yong Xia has suggested that elevated PCSK9 expression can activate the TLR4/myeloid differentiation primary response gene 88 (MyD88)/NF-κB and NLRP3 pathways, ultimately leading to inflammation and subsequent vascular endothelial dysfunction [66]. Moreover, oxLDL, a potent NF-κB activator, has been found to upregulate PCSK9 expression by stimulating the secretion of IL-6,IL-1α and TNF-α in both endothelial cells and macrophages [78]. Consistently, evolocumab can reduce inflammation by inhibiting the activation of TLR4 and nuclear NF-κB, as well as decreasing the levels of IL-1β and TNF-α [79]. And LPS can promote inflammation by inducing the expression of PCSK9 [40]. Mechanistically, it is suggested that LPS may upregulate PCSK9 expression by suppressing the transcription factors of farnesoid X receptor (FXR) and PPAR-α, which act as inhibitors of PCSK9 [80].

Within the realm of tissue engineering, tissue-engineered blood vessels (TEBVs) utilizing vascular interstitial smooth muscle cells (viSMCs) and vascular interstitial endothelial cells (viECs) that overexpress PCSK9 have shown a significant increase in viEC dysfunction, including an enhanced production of vascular cell adhesion molecule-1 (VCAM-1), TNF-α, and IL-6 when exposed to enzyme-modified LDL (eLDL) and TNF-α [81]. Furthermore, depletion of PCSK9 has been observed to impede the recruitment of macrophages and the mRNA expression of proinflammatory cytokines within aortic grafts [56].

Downregulation of PCSK9 has been linked to a decrease in endothelial fractalkine (CX3CL1) and C-X-C motif chemokine ligand 16 (CXCL16) production, as well as a reduction in the secretion of cytokines such as TNF-α, IL-1β, and interleukin-18 (IL-18) [24, 71]. Additionally, PCSK9 has demonstrated a suppressive effect on the proinflammatory response in VECs by inhibiting the uptake of lipopolysaccharide (LPS) through LDLR-mediated endocytosis, ultimately resulting in a decrease in IL-8 and IL-6 gene expression, VCAM-1/intercellular adhesion molecule-1 (ICAM-1), and protein secretion expression [82]. Extensive clinical data was gathered and examined from 27 patients with stable coronary heart disease and 27 healthy controls. And the analysis revealed a strong positive relationship between PCSK9 and vascular-related inflammatory factors, including endothelial selectin, interferon-gamma (IFN-γ), and IL-17 levels [83].

### 3.4 PCSK9 and vascular calcification

Vascular calcification (VC) is characterized by the abnormal deposition of calcium salts in vascular tissue, a phenomenon often associated with the aging of blood vessels [84]. In regions where calcification occurs, the protein PCSK9 is found to be highly expressed, particularly in VSMCs displaying a synthetic phenotype [85]. Studies involving both human and rat smooth muscle cells genetically engineered to produce increased levels of PCSK9 have demonstrated a notable increase in extracellular calcium accumulation when exposed to a procalcific environment. Treatment with PCSK9 resulted in heightened levels of pro-calcific markers such as alkaline phosphatase (ALP) and bone morphogenetic protein 2), while reducing the presence of anti-calcific factors like matrix GLA protein (MGP) and osteopontin [38]. Furthermore, the introduction of mutant mPCSK9 (D377Y) via AAV injection has been shown to induce vascular calcification in wild-type C57 mice [86]. Interestingly, the natural hypocholesterolemic agent Policosanol has been found to effectively inhibit the expression of calcification markers and attenuate the progression of aortic calcification by lowering PCSK9 levels in the bloodstream [87]. Clinical observations from a recent study have highlighted a potential link between high ankle-brachial index (ABI) values exceeding 1.4 and elevated serum concentrations of PCSK9, suggesting a possible involvement of PCSK9 in the development of vascular calcification among individuals with atrial fibrillation (AF) [88] (Fig. 1).

### 3.5 PCSK9 and immune senescence

Immunosenescence, an age-related deterioration of immunological processes, contributes to the heightened incidence and severity of infectious diseases, as well as the diminished efficacy of vaccinations in older individuals[89]. Immunosenescence is defined by a decline in cell-mediated immune activity and diminished humoral immunological responses [90, 91]. Clinical trials have established an association between PCSK9 and immunological response, notably in patients with disorders associated with vascular aging, such as atherosclerotic disease and coronary artery disease [92-94]. PCSK9 could serve as a potential key regulator of T-cell activation and expansion. Additionally, it may play a role in modulating inflammation and influencing the activity of other immune cell types [95]. PCSK9 can diminish LDLR levels and T cell receptor (TCR) signaling in CD8+ T cells, obstructing the recycling of LDLR and TCR to the plasma membrane, therefore impeding the effector function of CTLs [96]. PCSK9 markedly diminished the expression of the member 1 of human transporter sub-family ABCA (ABCA1) gene in WT macrophages (decreased by 64%, p < 0.001), which substantially impacted TCR signaling in CD8+ T cells, hence reducing T cell survival, proliferation, and differentiation [97, 98].

## 4. PCSK9 and aging-related vascular disease

**Figure 3.**
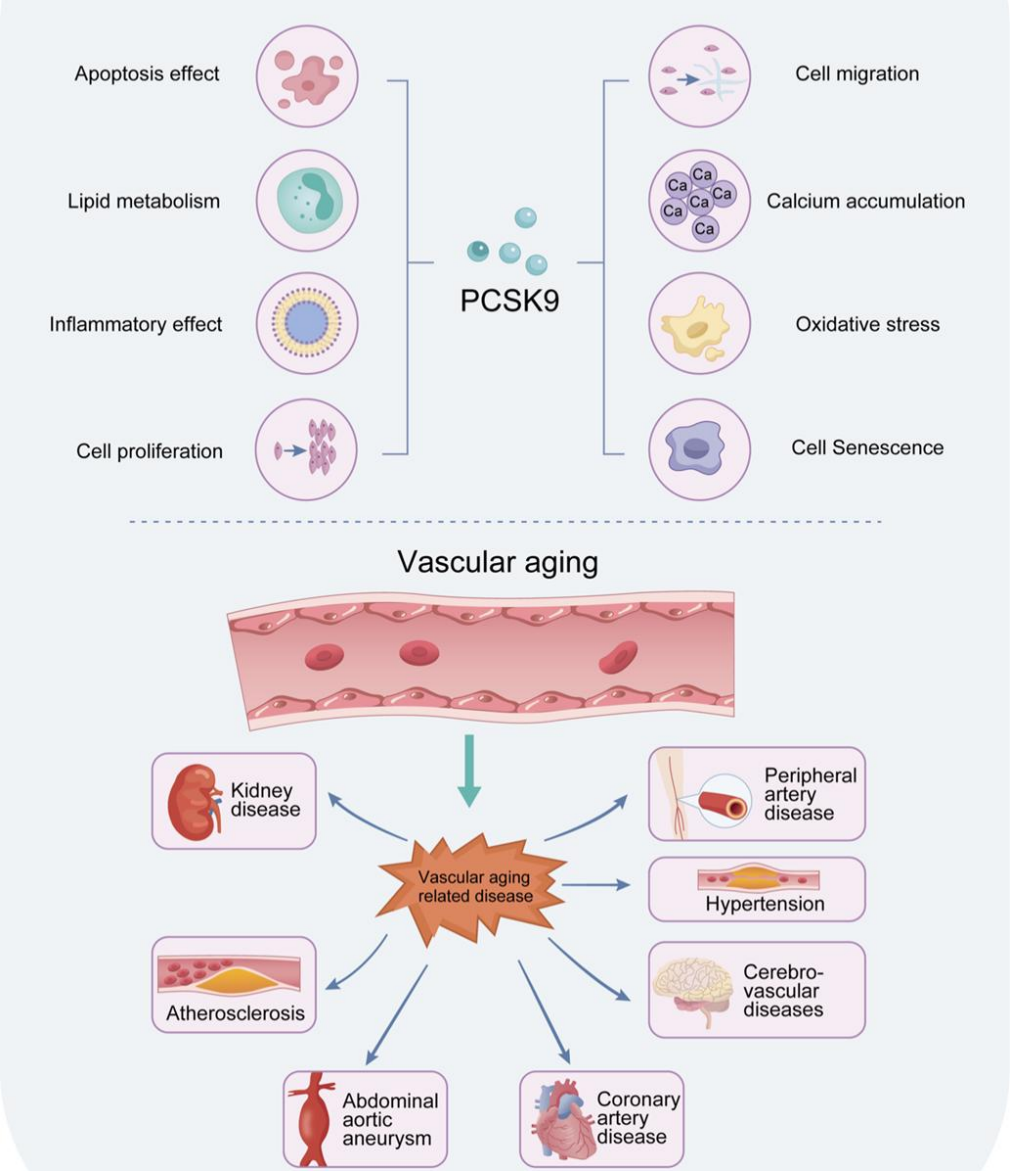
**PCSK9 and Vascular Aging-Related Diseases**. PCSK9 influences vascular aging through lipid metabolism and processes such as cellular senescence, inflammation, apoptosis, proliferation and migration, oxidative stress, and extracellular matrix calcification. These effects contribute to the development and progression of vascular aging-related diseases, including atherosclerosis, hypertension, abdominal aortic aneurysm, coronary artery disease, peripheral artery disease, cerebrovascular disease, and kidney diseases.

Recent research has brought to light the pivotal role of PCSK9 in various aging-related cardiovascular diseases [36, 52]. In the context of atherosclerosis, PCSK9 emerges as a key player in the development and progression of degenerative vascular disease, particularly in coronary arteries, by influencing LDL cholesterol levels and inflammation [99]. Evidence suggests that inhibiting PCSK9 can mitigate endothelial dysfunction and reduce the size of atherosclerotic lesions, offering a promising avenue for therapeutic interventions in atherosclerotic diseases [100]. Furthermore, PCSK9's involvement in atherosclerosis extends beyond LDL cholesterol regulation, impacting vascular inflammation and apoptosis of endothelial cells [25, 37]. Moving beyond atherosclerosis, PCSK9's implications in abdominal aortic aneurysm (AAA) and coronary artery disease (CAD) bring to light its viability as a therapeutic target in addressing inflammatory processes and cardiovascular risks associated with these conditions [40, 101-103]. Additionally, PCSK9's influence on hypertension, peripheral artery disease, and kidney diseases underscores its multifaceted role in vascular health and its promise as a target for mitigating a range of cardiovascular complications [59, 104-106] (Fig. 3) (Table 1). As research continues to unveil the intricate connections between PCSK9 and various vascular diseases, the potential for developing targeted therapies aimed at PCSK9 inhibition opens new avenues for addressing complex cardiovascular conditions.

**Table 1:** Roles of PCSK9 in vascular aging related diseases and related medicine.

	Disease	Functions	Reference
**Cardiovascular diseases**	AS AAA CAD AMI	Control LDL cholesterol levels; suppress vascular inflammation; Regulate LEA, inflammatory signaling pathways, histone deacetylase SIRT1 and NF-κB pathways; Control levels of Lp(a); Regulate TLR4/MyD88/NF-κB axis; Bnip3-mediated autophagic pathway.	[110, 111, 188] [67, 189] [124, 125, 190] [127, 129]
**Cerebrovascular diseases**	AIS	May be independent of LDL-C and Lp(a) levels	[135]
**Hypertension**	EH	Control the movement of the ENaC protein to the cell surface; influence the breakdown of proteins in the proteasome	[141, 143]
**Peripheral artery disease**	PAD	Regardless of other lipid parameters and conventional cardiovascular risk factors	[148]
**Kidney Diseases**	CKD	Enhanced expression of the Hnf1α gene and its target genes	[152, 191]

AS: atherosclerosis AAA: abdominal aortic aneurysm; LEA: Leukocyte-endothelium adhesion; CAD: coronary artery disease; AMI: acute myocardial infarction; EH: essential hypertension; AIS: acute ischemic stroke; CKD: chronic kidney disease; PAD: peripheral artery disease; LDL: low-density lipoprotein NF-κB: nuclear factor kappa-light-chain-enhancer of activated B cells; TLR4: toll-like receptor 4; SIRT1: sirtuin 1.

### 4.1 PCSK9 and atherosclerosis

PCSK9 has been identified in the VSMCs from human atherosclerotic lesions, underscoring its significance in the development of degenerative vascular disease [107]. Additionally, studies have shown that the concentration of PCSK9 in plasma is elevated in atherosclerotic plaques, further highlighting its prospective function in the progression of atherosclerosis [108]. In a study involving PCSK9 D374Y transgenic minipigs with elevated cholesterol levels, researchers observed a notable increase in coronary atherosclerosis and the persistent formation of sizable fibroatheromas, leading to cephalad hypertension. This suggests a direct link between PCSK9 and the development of atherosclerotic lesions in the coronary arteries. Inhibition of PCSK9 has also been shown to reduce endothelial dysfunction and the size of atherosclerotic lesions, suggesting a potential therapeutic target for the treatment of atherosclerotic diseases [109] (Fig. 3).

PCSK9 is a crucial regulator of atherosclerosis by controlling LDL-C levels. Atherosclerosis, a major contributor to age-related cardiovascular diseases (CVD), is characterized by the accumulation of LDL-C in blood vessels. Individuals with loss-of-function (LOF) mutations in PCSK9 exhibit higher levels of liver LDLR, leading to lower LDL-C levels and a 50 to 86% decrease in the lifetime risk of CAD relative to non-mutation bearers [110]. Human VSMCs release physiologically active PCSK9, which significantly reduces LDLR expression in human macrophages and other peripheral cells. This underscores the critical role of PCSK9 in the development and progression of atherosclerosis, with potential implications for therapeutic interventions targeting this pathway [20].

PCSK9 affects atherosclerosis in an LDL-independent manner. Studies have shown that PCSK9 directly enhances atherosclerotic lesions through an LDL-independent mechanism, leading to vascular inflammation and atherosclerosis [111]. PCSK9 activates PKCδ, Syk and NF-κB, promoting atherosclerosis progression independently of LDL-R [112].In apoE^-/-^ mice, the inactivation of PCSK9 has been shown to effectively suppress vascular inflammation and atherosclerosis by reducing the TLR4/NF-κB signaling pathway [113, 114]. Ox-LDL is known to contribute significantly to the accumulation of cholesterol esters in macrophages VSMC and macrophages [115]. The buildup of ox-LDL particles in artery walls is mediated by LOX-1, which is increased in reaction to inflammatory stimuli [30]. Interestingly, a positive feedback loop exists between PCSK9 and LOX-1 in the context of inflammation inside vascular tissue., where PCSK9 activates LOX-1 and vice versa [21]. Furthermore, PCSK9 has been found to directly influence the development of atherosclerosis by affecting the apoptosis of ECs [69]. In HUVEC, PCSK9 knockdown has demonstrated the ability to avert ox-LDL-induced apoptosis by blocking the activation of caspase 3 and 9[70]. In a mouse model prone to atherosclerosis, deletion of the PCSK9 gene, independent of LDLR, reduced the production of adhesion molecules and chemotactic factors from endothelial cells, such as ICAM-1, monocyte chemoattractant protein-1 (MCP-1), and monocyte chemoattractant protein-3 (MCP-3), which facilitate monocyte adhesion and penetration into vascular walls [116].

### 4.2 PCSK9 and abdominal aortic aneurysm

AAA is a complex vascular disease characterized by the weakening and dilation of the abdominal aorta [117]. Recent studies have emphasized the possible involvement of PCSK9 in the onset and advancement of AAA. Studies have shown that PCSK9 expression is increased in AAA tissues and is closely associated with macrophages, indicating its involvement in the inflammatory process underlying AAA [101]. In animal models of AAA induced by hyperlipidemia and angiotensin II, deletion or inhibition of PCSK9 has been found to significantly reduce the formation of AAA lesions, improve survival rates, and decrease systemic inflammation, independent of changes in circulating cholesterol levels [101, 118]. Furthermore, PCSK9 deficiency has been shown to reduce macrophage infiltration into the aortic wall, prevent elastin degradation, and suppress the expression of proinflammatory cytokines and matrix metalloproteinase 9 (MMP9) [101]. The molecular mechanisms underlying these effects involve the modulation of leukocyte-endothelium adhesion, inflammatory signaling pathways, and the regulation of histone deacetylase sirtuin 1 (SIRT1) and NF-κB pathways [67]. Importantly, PCSK9 inhibitors have shown promise in limiting AAA progression and vascular inflammation, suggesting their potential as a targeted therapy for this condition. Genetic studies and Mendelian randomization analyses have further supported the role of PCSK9 in AAA pathogenesis and identified the opportunity for repurposing PCSK9 inhibitors to mitigate AAA growth and reduce cardiovascular risks in affected individuals [119, 120].

The emerging evidence indicates that targeting PCSK9 may offer a promising therapeutic strategy for treating AAA by addressing the inflammatory processes, macrophage activation, and elastin degradation associated with the disease. Ongoing research, including clinical studies, is essential to substantiate the efficacy and safety of PCSK9 inhibition in the management of AAA and its associated cardiovascular complications (Fig. 3).

### 4.3 PCSK9 and coronary artery disease

PCSK9, a gene associated with CAD, has been the focus of numerous studies on its role in cardiovascular health. Research has shown that individuals carrying the G allele of PCSK9 are more susceptible to CAD [121, 122]. Furthermore, elevated levels of PCSK9 have been linked to an increased risk of CAD and subsequent cardiovascular events [123, 124]. Research has indicated a direct association between PCSK9 levels in the bloodstream and the likelihood of developing CAD [124]. Through genetic inhibition of PCSK9, researchers have observed a decrease in CAD risk due to reduced levels of lipoprotein(a) (Lp(a)) [125]. In addition to genetic factors, the aging process has been identified as a major factor in the development of CAD following an acute myocardial infarction (AMI). Clearance of senescent cells can improve diastolic function, myocardial remodeling and overall survival post-MI [126]. Moreover, research has shown that increased expression of PCSK9 is associated with impaired cardiac function post-AMI. Elimination of PCSK9 has resulted in reduced infarct size, improved cardiac function, and decreased inflammatory cell infiltration in animal models. The activation of the TLR4/MyD88/NF-κB axis has been proposed as a potential mechanism for this effect [127, 128]. Additionally, PCSK9 knockdown has been shown to mitigate myocardial infarction-related injury by activating the Bnip3-mediated autophagic pathway, suppressing the inflammatory response, and protecting heart function [129]. Studies have also highlighted the transient upregulation of PCSK9 in the acute phase of AMI, driven by transcription factors SREBP-2 and HNF1α [130]. Furthermore, the administration of alirocumab to AMI patients for one year has led to a significant decrease in angiographic stenosis percentage [131]. A predetermined secondary analysis of the evaluation of cardiovascular outcomes after an acute coronary syndrome when treatment with alirocumab (ODYSSEY OUTCOMES) trial demonstrated that the beneficial effects of the PCSK9 inhibitor alirocumab were significantly extended to the older patients involved in the study[132] (Fig. 3)

### 4.4 PCSK9 and cerebrovascular diseases

Arterial stiffness, a common feature of vascular aging, is associated with an increased risk of stroke, particularly fatal stroke in individuals with hypertension [133]. Recent studies have suggested that the beneficial effects of PCSK9 inhibitors on stroke risk may be independent of LDL-C and Lp(a) levels [134]. PCSK9 inhibitors have demonstrated efficacy to reduce the risk of ischemic stroke. For example, the addition of evolocumab to statin therapy resulted in a decreased risk of ischemic stroke in patients with previous strokes who were treated with PCSK9 inhibitors [127, 135]. Additionally, lower levels of PCSK9 have been associated with poor outcomes and an increased risk of major adverse cardiovascular events following AIS[136, 137] (Fig. 3).

### 4.5 PCSK9 and hypertension

Hypertension, a condition characterized by high blood pressure, is closely linked to early vascular aging, a process that involves the deterioration of blood vessels [138, 139]. Fibrosis, perivascular inflammation, and vascular calcification are key factors in the development of hypertension, ultimately leading to vascular stiffening [140]. Research by sharotri highlighted the role of PCSK9 in regulating blood pressure in animal models. This study revealed that the PCSK9 gene product hinders the movement of the epithelial sodium channel (ENaC) protein to the cell surface, which is responsible for sodium absorption in the kidney and assumes a vital function in blood pressure regulation [141, 142]. By increasing the breakdown of proteins in the proteasome, PCSK9 influences sodium absorption levels and, consequently, blood pressure. Additionally, the inhibition of sterol regulatory element-binding protein-2 (Srebp-2) by fibroblast growth factor 21 (FGF21) triggers the secretion of corticosterone in the hypothalamus, promoting the synthesis of adiponectin in adipocytes [143]. Adiponectin, an adipokine known for its antihypertensive properties, further underscores the potential of PCSK9 as an early biomarker for detecting hypertension [144] (Fig. 3).

### 4.6 PCSK9 and peripheral artery disease

The term peripheral artery disease (PAD) traditionally includes the range of diseases that impact noncardiac, nonintracranial arteries [145]. The prevalence and incidence of PAD are significantly correlated with age, exceeding 10% in patients aged 60 and 70[146]. Elevated levels of PCSK9 were observed in individuals with PAD, regardless of other lipid parameters and conventional cardiovascular risk factors [147]. Alirocumab reduces the occurrence of PAD events in individuals with recent acute coronary syndrome who are on statins, particularly those with high Lp (a) [124, 148] (Fig. 3).

### 4.7 PCSK9 and kidney diseases

Chronic kidney disease (CKD) exhibits early vascular aging (EVA) [149, 150].The independent association between LDL-C and the advancement of CKD to end-stage renal disease [151]. Elevated PCSK9 levels in nephrotic syndrome correlate positively with proteinuria [105]. And it may be is related with the increased hepatic expression of the Hnf1α gene and its associated target genes [152]. Induction of uremic state in rats through dietary means resulted in elevated levels of Pcsk9 and total cholesterol in the plasma, livers, and kidneys [38] (Fig. 3).

## 5. PCSK9 and other aging-related diseases

Recent reports suggest that PCSK9 plays a crucial role in the onset and progression of many aging-related diseases, such as Alzheimer’s disease, cancer, and diabetes [153, 154].

### 5.1 PCSK9 and neurodegenerative disorders

Alzheimer’s disease (AD) is the most common form of dementia and can be defined as a gradually progressive neurodegenerative disorder characterized by the accumulation of amyloid-beta peptide Aβ [155]. Cholesterol is an essential chemical implicated in brain development. Changes in neuronal cholesterol levels influence Aβ metabolism and lead to neurodegeneration[156]. PCSK9 is acknowledged to perform various critical functions in the brain, including the regulation of neuronal differentiation, apoptosis, and, significantly, the functionality of LDL receptors. Furthermore, PCSK9 seems to be directly implicated in key processes associated with the development of Alzheimer's disease, such as inflammation, oxidative stress, and Aβ deposition [154]. PCSK9 may be activated by elevated ox-LDL levels in the brain linked to hyperlipidemia, hence facilitating neuronal apoptosis via the NF-κB-Bcl-2/Bax-caspase 9-caspase 3 signaling pathways [157]. Alirocumab likely mitigates high fat cholesterol diet (HFCD)-induced Alzheimer's disease-like conditions by inhibiting Aβ aggregation, HMGB1/RAGE/TLR4, and consequently glutamate levels [156]. Furthermore, prolonged ethanol exposure in male rats elevated PCSK9 expression in the brain, whereas treatment with Alirocumab markedly enhanced neuronal LDLR and diminished oxidative stress in both neurons and the cerebral vasculature [158]. PCSK9 inhibitors were found to alleviate cognitive dysfunction in type 2 diabetes mellitus (T2DM) rats by reducing the expression of PCSK9, IL-1β, IL-6, and TNF-α while increasing LDLR expression in hippocampal tissues [159].

### 5.2 PCSK9 and cancer

The correlation between cancer prevalence and age is generally recognized within the medical field. The majority of significant tumors are more prevalent in males and women aged over 65 years [160]. The interplay between PCSK9 and cholesterol homeostasis may impact membrane dynamics and cellular migration, thereby further influencing tumor aggressiveness [161]. The broad cellular processes by which PCSK9 affects cancer progression are not limited to lipid regulation. Recent studies have demonstrated that PCSK9 can regulate immune responses through interactions with immune cells and components of the tumor microenvironment. These include effects on dendritic cell maturation, T cell activation, and cytokine production, suggesting a role in shaping antitumor immune responses [162]. Eliminating the PCSK9 gene in murine cancer cells markedly suppresses their proliferation in a way reliant on cytotoxic T lymphocytes. This gene deletion also improves the effectiveness of immune treatment aimed at the checkpoint protein PD1. Inhibition of PCSK9 enhances the expression of major histocompatibility complex class I (MHC I) proteins on tumor cell surfaces, facilitating significant intratumoral infiltration of cytotoxic T lymphocytes [163].

**Table 2:** Main new research findings: Clinical trials investigating PCSK9 inhibitors.

Population	Trial phase	Drug type	Outcomes	Limitations	Ref.
**Healthy volunteers with an LDL-C level of at least 100 mg per deciliter**	Phase 1	Long-acting RNAi therapeutic agent: Inclisiran	With a 50.6% reduction of LDL-C levels observed in the group receiving 500 mg of Inclisiran (p<0.001; 95% CI 36.96-61.3)	Cough; musculoskeletal pain; nasopharyngitis; headache; back pain and diarrhea.	[192]
**36 healthy participants with LDL-C ≥70 mg/dL and ≤190 mg/dL**	Phase 1	oral small molecule PCSK9 inhibitor: AZD0780	AZD0780 60 mg as monotherapy reduced LDL-C 38% (p<0.001; 95%CI 30-48) compared to baseline	No serious adverse events	[193]
**110 patients receiving stable dose of atorvastatin with an LDL-C level of 2.6 mmol/L**	Phase 1b/2	PCSK9 inhibitor: recaticimab	In 450 mg Q12W, With a 52.77% reduction of LDL-C (p<0.001; 95% CI 45.05-60.49)	Upper respiratory tract infection;increased gamma glutamyl transferase	[194]
**267 patients with confirmed ASCVD (aged ≥40 years) or at elevated risk of ASCVD**	Phase 2	Orally administered NNC0385-0434	Decrease in LDL-C from baseline to week 12, with a drop of 61.8% (p<0.0001; 95%CI 50.7 to 72.9)	Gastrointestinal issues	[195]
**375 adult participants with a wide range of ASCVD risks.**	Phase 2b	A Novel Oral Macrocyclic Peptide: MK-0616	At Week 8, MK-0616 demonstrated a reduction in LDL-C of up to 60.9% (95% CI 67.6-54.3, P < 0.001)	COVID-19 infection; arthralgia	[196]
**50 eligible individuals (≥12 years) with homozygous familial hypercholesterolemia**	Phase 3	Anti-PCSK9 antibodies: evolocumab	Reduce ultracentrifugation LDL-C at 12 weeks by 30.9% (p<0.0001; 95%CI 18-43.9)	Muscle complaints	[197]
**223 adult patients with hypercholesterolemia**	Phase 3	Anti-PCSK9 antibodies: alirocumab	with alirocumab 150Q4W, resulting in LDL-C decreases of 51.7% at week 24 (p<0.0001)	Muscle-related symptoms	[198]
**18,924 patients with a history of ACS in the 1-12 months**	Phase 3	Anti-PCSK9 antibodies: alirocumab	The risk of death from ACS, MI, IS, or unstable angina decreased by 16.2% (95% CI 5.5-26.8, P=0.002, HR 0.06).	Local injection-site reactions	[199]
**27,564 patients with ASCVD with an LDL > 70 mg/dL or higher**	Phase 3	Anti-PCSK9 antibodies: evolocumab	An LDL-C reduction of 59% (P<0.001, 95% CI 58-60) compared to the baseline and the risk of AIS (9.8% vs 11.3%) (HR 0.85, 95% CI 0.79-0.92, P<0.001)	Exception of injection-site reactions	[200]
**3642 patients with confirmed PAD**	Phase 3	Anti-PCSK9 antibodies: evolocumab	Reduced the risk of MALE in all patients (HR 0.58, 95% CI 0.38-0.88, P=0.0093)	No excess of adverse events	[201]
**8,077 patients with preserved kidney function, 15,034 with stage 2 CKD, and 4,443 with ≥stage 3 CKD**	Phase 3	Anti-PCSK9 antibodies: evolocumab	Relative risk reduction for preserved function (HR: 0.82; 95% CI: 0.71 to 0.94), stage 2 (HR: 0.85; 95% CI: 0.77 to 0.94), and stage ≥3 CKD (HR: 0.89; 95% CI: 0.76 to 1.05)	Estimated glomerular filtration rate decline	[191]

LDL-C: low-density lipoprotein cholesterol; ASCVD: atherosclerotic cardiovascular disease; ACS: acute coronary syndrome; MI: myocardial infarction; IS: ischemic stroke; UA: unstable angina; PAD: peripheral artery disease; MALE: major adverse limb events; CKD: chronic kidney disease; HR: hazard ratio.

## 6. Pharmacological approaches to target PCSK9

Lowering plasma total cholesterol and low-density lipoprotein cholesterol (LDL-C) significantly enhances cardiovascular health, therefore alleviating the effects of age-related diseases [164]. The targeting of PCSK9 has emerged as a pivotal therapeutic strategy in the management of hypercholesterolemia and cardiovascular disease [165]. As research elucidates the multifaceted roles of PCSK9 in lipid metabolism, several pharmacological approaches have been developed to inhibit its function, with varying degrees of clinical success and implications for patient care [166] (Table 2). Preclinical research has provided valuable insights into the biological functions of PCSK9 [167, 168] (Table3).

**Table 3:** Main new research findings: basic research between PCSK9-related drugs and age-related vascular diseases.

Research subject	Drug type	Main Findings	Ref.
**C57 mice with AAA**	Anti-PCSK9 antibodies: evolocumab	Suppresses AAA progression in mice	[189]
**Aging rats**	PCSK9 inhibition with alirocumab	effectively reduced the CVD progression	[52]
**Male Wistar rats with** **cardiac I/R injury**	PCSK9 inhibitor: pep2-8	Give prior to ischemia exerted cardio protection through protection of cardiac mitochondrial function, decreased infarct size and improved LV function	[202]
**Mice exposed to chronic hypoxia (10%)**	PCSK9 monoclonal antibody	Hypoxia plus SU5416-induced PAH was attenuated	[59]
**ApoE^-/-^ mice and human PCSK9 D374Y overexpression mice with AS**	A small-molecule PCSK9 inhibitor: E28362	Significantly decrease plasma LDL-C levels and the area of atherosclerotic lesions in enface aortas and aortic roots.	[203]

AAA: abdominal aortic aneurysm; CVD: Cardiovascular Disease; I/R: ischemia/reperfusion; LV: left ventricular; PAH: pulmonary arterial hypertension; LDL-R: low-density lipoprotein receptor; AS: atherosclerosis.

### 6.1 Basic to translational science of PCSK9 inhibitors

Studies utilizing PCSK9 gene knockout models have shown that the absence of this protein leads to a reduction in circulating cholesterol levels [169, 170]. However, the translation of these findings to human applications is challenging due to the inherent physiological differences between species. Alba Carreras conducted a pivotal work addressing the deficiency of validated models for assessing the safety and efficacy of treatments aimed at human PCSK9. Alba Carreras created a liver-specific human PCSK9 knock-in mouse model (HPCSK9-KI). The plasma concentration of total cholesterol in HPCSK9-KI mice was observed to be elevated compared to wild-type mice and increased with age. Treatment with evolocumab, a monoclonal antibody that targets human PCSK9, decreased cholesterol levels in HPCSK9-KI mice but not in wild-type mice, suggesting that the hypercholesterolemic phenotype was induced by the overexpression of human PCSK9. CRISPR-Cas9-mediated genome editing of human PCSK9 diminished plasma concentrations of human PCSK9, while leaving mouse PCSK9 unaffected, hence resulting in a reduction of total plasma cholesterol levels. Genome editing of mouse PCSK9 failed to decrease cholesterol levels. Base editing employing guide RNAs directed at both human and murine PCSK9 lowered plasma concentrations of human and murine PCSK9, in addition to total cholesterol levels[171]. Recent evidence suggests that individuals with loss-of-function mutations in PCSK9 have a lower risk of cardiovascular events [172, 173], highlighting the role of PCSK9 in the pathogenesis of age-associated CVD. In both people and rats, blood levels of PCSK9 exhibited a favorable correlation with aging and the incidence of cardiovascular disease. Moreover, the suppression of PCSK9 using Alirocumab significantly decelerated the advancement of cardiovascular disease in aging rats, suggesting that PCSK9 is pivotal in cardiovascular aging [52]. However, it is important to note that these individuals may also have an increased susceptibility to infections, indicating a complex role of PCSK9 in immune response [174, 175]. This nuanced aspect of PCSK9 function is not fully captured in animal models and underscores the need for further research to fully understand the implications of PCSK9 modulation in humans.

### 6.2 The limitations of PCSK9 inhibitors

Pharmacological strategies to inhibit PCSK9 primarily target its synthesis and function. These strategies include gene silencing agents, monoclonal antibodies (mAbs) that prevent PCSK9 from binding to the LDLR, and small-molecule inhibitors that block PCSK9 autocatalytic processing [176]. Clinical trials, such as those evaluating evolocumab and alirocumab, have unequivocally demonstrated that PCSK9 inhibition effectively lowers LDL cholesterol levels and reduces cardiovascular event risk. Nevertheless, patient safety remains a paramount concern [177]. The frequently reported adverse effects of PCSK9 inhibitors in clinical practice encompass myalgia (27.2%), back pain (12%), nasopharyngitis (9.3%), headache (9.2%), upper respiratory tract infections (9%), flu-like symptoms (7.5%), and joint pain (7%)., with a notable incidence of myalgia in post-marketing surveillance studies [174, 178]. T Cardiovascular medicine is striving to study PCSK9 inhibitor drugs, not primarily due to concerns about efficacy or safety, but because the annual cost of these new drugs exceeds $14,000—more than 100 times higher than that of generic statins [179]. Only a small fraction of this substantial cost is expected to be offset by the prevention of cardiovascular events. Currently, the cost-effectiveness of PCSK9 inhibitors falls short of widely accepted standards for value in the U.S. High out-of-pocket expenses for PCSK9 inhibitors pose significant barriers to patient access and may adversely impact long-term adherence. In addition, the need for subcutaneous injections every two weeks or monthly for PCSK9-targeting human monoclonal antibodies such as evolocumab and alirocumab has raised concerns about patient adherence [180]. Additionally, if all eligible patients were to receive these treatments, the financial burden on healthcare systems would be considerable, raising critical challenges for policymakers, insurers, and society at large [181]. Moreover, Ongoing studies like the EPIC-HIV trial are delving deeper into the therapeutic possibilities of PCSK9 inhibitors in various clinical settings [182, 183]. The widespread adoption of these therapies is not without its ethical and economic challenges. The cost of PCSK9 inhibitors presents a significant barrier, potentially restricting access to advanced therapies [181]. Additionally, ethical considerations surrounding these treatments must be carefully weighed against potential adverse effects and the patient's overall health status.

The efficacy of PCSK9 inhibitors shows promise, but there is a need for tailored therapy due to the variability in treatment outcomes among different patient populations[184]. For instance, the CREDIT-4 trial demonstrated significant LDL-C reduction with tafolecimab, while the ORION-1 trial revealed less prominent effects with inclisiran [185, 186]. In one study, participants in PCSK9 inhibitor trials were recruited worldwide, and seven meta-analyses encompassing 376,885 patients indicated substantial treatment heterogeneity, as seen by an I² mean of 48% across outcomes, with two analyses concentrating on primary outcomes of all-cause death [187]. This diversity highlights the significance of considering patient-specific factors, such as individual tolerability and underlying health conditions, when selecting suitable therapies.

## Conclusion

In conclusion, aging is a natural biological process that contributes significantly to the development and progression of vascular related diseases, which are major causes of mortality worldwide. Understanding the mechanisms of arterial aging is crucial for addressing age-related vascular disorders and implementing anti-aging interventions to manage chronic cardiovascular conditions in the elderly.

PCSK9, known for its role in regulating LDL cholesterol levels, has been implicated in various metabolic processes linked to vascular aging. Studies have shown that PCSK9 has a significant impact on vascular cell senescence, proliferation, migration, apoptosis, inflammation, calcification, and the development of aging-related vascular diseases. By affecting processes such as VSMC and endothelial dysfunction, cellular senescence, and vascular inflammation, PCSK9 plays a multifaceted role in vascular aging and the pathogenesis of vascular diseases. Furthermore, the therapeutic potential of PCSK9 inhibitors in treating vascular aging and related diseases has garnered attention, highlighting the importance of understanding the biological characteristics and mechanisms of action of PCSK9 in the vascular system. Collaboration and further research in this area may lead to the development of novel therapeutic interventions targeting PCSK9 to address age-related cardiovascular disorders and improve vascular health.

Exploring the role of PCSK9 in aging-related vascular diseases provides valuable insights into the molecular mechanisms underlying vascular aging and offers potential avenues for therapeutic interventions to mitigate the impact of age-related cardiovascular conditions. By elucidating the intricate interplay between PCSK9 and vascular health, researchers can pave the way for advancements in managing and treating aging-related vascular diseases, ultimately improving the quality of life for aging populations.

The development of PCSK9 inhibitors has become a pivotal strategy in managing hypercholesterolemia and cardiovascular diseases. Although PCSK9 inhibitors hold great promise, their economic and ethical challenges must be addressed to enable broader clinical application.
